# Roasting-driven flavor reconstruction in Wuyi Rougui tea: a dual mechanism of pectin-mediated thickening and metabolite remodeling

**DOI:** 10.3389/fpls.2026.1938406

**Published:** 2026-08-28

**Authors:** Hua Feng, Jiawei Yan, Yiting Liu, Xiaohong Feng, Ruihua Liu, Fang Yang, Yanhua Zhou, Yao Wu, Yushan Liu, Jiahua Chen, Xuchao Yin, Weiguo Zhao, Feiquan Wang, Pengjie Wang, Zongjie Wu

**Affiliations:** 1 College of Tea and Food Science/Tea Engineering Research Center of Fujian Higher Education/Tea Science Research Institute, Wuyi University, Wuyishan, China; 2 School of Modern Agriculture and Biotechnology, Ankang University/Shaanxi Tea Lab Co-constructed by Province and Municipality, Ankang, China; 3 College of Horticulture, Northwest A&F University, Xianyang, China; 4 Fujian Keycaa Biotechnology Co. Ltd, Xiamen, China; 5 Minbei Vocational and Technical College, Nanping, China

**Keywords:** metabolomics, molecular docking, roasting process, sensory omics, Wuyi Rougui

## Abstract

Roasting shapes the sensory quality of Wuyi Rougui tea, but the links between sensory changes and flavor compound remodeling across roasting stages remain insufficiently characterized. Sensory evaluation and electronic sensory analysis showed that RG2O exhibited enhanced sensory characteristics, including fruity-floral and cinnamon-like aroma attributes. GC-MS analysis identified 362 volatile metabolites, with esters, terpenoids, and heterocyclic compounds as the major classes. OAV and VIP analyses suggested that 1-nonen-3-one, pyrazines, and other differential volatiles may contribute to the variation of mineral and roasted aroma characteristics. Non-volatile analysis showed that gallic acid (GA), gallocatechin gallate (GCG), and theabrownins were associated with taste-related attributes, including thickness, while roasting was accompanied by the conversion of protopectin into soluble pectin. Molecular docking provided predictive evidence for potential interactions between GA/GCG and the calcium-sensing receptor related to kokumi-associated perception. Overall, roasting was associated with coordinated remodeling of volatile and non-volatile metabolites, together with changes in pectin components, contributing to the reconstruction of flavor characteristics in Wuyi Rougui tea.

## 1 Introduction

Tea (*Camellia sinensis*), the most widely consumed non-alcoholic beverage worldwide, has attracted considerable attention due to its distinctive flavor profile and health-promoting properties (Qiao et al., 2025; Lei et al., 2026a). Wuyi Rock Tea, produced in the Wuyi Mountains of Fujian Province, China, is a semi-fermented oolong tea renowned for its characteristic “rock charm” and unique sensory quality (Zeng et al., 2020). This “rock charm” is typically manifested as a full-bodied taste, a mineral-like note, and a lingering sweet aftertaste, yet its precise chemical basis remains largely unclear. Among oolong tea cultivars, Rougui is considered a representative variety due to its rich aroma, robust flavor, and pronounced cinnamon-like spicy note (Wang et al., 2025; Lei et al., 2026b). The formation of these sensory characteristics depends on a diverse pool of metabolites and biochemical precursors, including catechins, free amino acids, and glycosidically bound volatile precursors, which are accumulated during tea plant growth and preliminary processing steps such as withering and green-making (Lai et al., 2025). These complex biochemical substrates undergo further refinement through roasting, a critical step that significantly affects the flavor and quality grade of the final tea product (Zhan et al., 2023). Roasting involves controlled thermal treatment that triggers extensive metabolic and physicochemical transformations within tea leaves, not only enhancing physical stability by reducing water activity but also promoting the mellowing of the tea infusion and the maturation of aroma through thermochemical reactions (Wang et al., 2022). Previous studies have indicated that metabolite alterations induced by roasting are more pronounced than those occurring during other processing steps, highlighting its pivotal role in flavor reconstruction (Liu et al., 2022). However, the dynamic changes in tea metabolites during roasting and their associations with sensory quality formation remain highly complex, and sensory experience alone is insufficient to elucidate their chemical essence. Therefore, a systematic investigation of roasting-induced chemical transformations in Wuyi rock tea is essential for understanding flavor development and supporting the standardized production and targeted quality control of rock teas.

Previous studies on Wuyi rock tea roasting have mainly focused on changes in volatile compounds and have demonstrated the importance of the Maillard reaction, Strecker degradation, lipid oxidation, and glycoside hydrolysis in the formation of roasted aroma characteristics (Yang et al., 2023). By comparison, the chemical and physical basis of “thickness”, an important mouthfeel attribute of Wuyi rock tea, has received considerably less attention. The ratio of polyphenols to amino acids has frequently been used as an indicator of the balance among bitterness, astringency, freshness, and overall taste coordination. However, this ratio alone cannot fully account for the complex mouthfeel changes induced by roasting. In particular, the effects of thermal treatment on cell wall polysaccharides and their potential contributions to the viscosity, smoothness, and fullness of tea infusions remain insufficiently understood (Hirono and Mizukami, 2018; Zhou et al., 2021). Moreover, metabolomic analysis can identify roasting-associated changes in chemical composition, whereas conventional correlation analysis does not directly resolve potential interactions between flavor-active compounds and sensory receptors. In this study, we distinguish between the sensory attributes of “thickness” and “kokumi”. “Thickness” primarily reflects the physical viscosity and oral tactile properties of the tea infusion and may be associated with the rheological behavior of macromolecular components such as soluble pectins. In contrast, “kokumi” describes a sensation that does not generate a distinct primary taste but may enhance the persistence, fullness, and complexity of other taste sensations, and has been associated with calcium-sensing receptor (CaSR)-mediated signaling (Kuroda and Miyamura, 2015). This distinction provides a framework for considering flavor reconstruction from two complementary perspectives: pectin-associated physical thickening and receptor-related chemical modulation.

With the advancement of intelligent sensory technologies, the integration of sensory evaluation and metabolomics has been further strengthened (Lu et al., 2024). Specifically, computer vision (electronic eye) is applied to digitally characterize the dynamic changes in tea infusion appearance and color, whereas electronic tongue and electronic nose systems capture gustatory and olfactory features, respectively. Drawing on an integrated sensory–metabolomic strategy in tea quality research (Li et al., 2025), this study selected samples representing four typical roasting stages to systematically investigate the changes in characteristic aroma and taste compounds during roasting of Wuyi Rougui. Additionally, we examined the thermal transformation of pectin components and the potential contribution of roasting-induced β-elimination reactions to the thickness and smoothness of the tea infusion. Molecular docking analyses were further used to predict the potential binding modes of key flavor molecules with the olfactory receptor OR1A1 and CaSR. Collectively, this work aims to provide insights into the potential mechanisms of flavor reconstruction in Wuyi Rougui, providing a theoretical foundation for optimizing rock tea processing and supporting digital quality evaluation.

## 2 Materials and methods

### 2.1 Sample collection

Fresh leaves of ‘Rougui’, consisting of one bud with four to five leaves, were harvested from Niuyang Tea Plantation, Yangzhuang Township, Wuyishan City, Fujian Province, China. Under the same equipment and environmental conditions, preliminary tea samples were prepared following the conventional oolong tea processing steps, including withering, green-making, pan-firing, rolling, and drying. The obtained preliminary tea samples were used as the starting material for subsequent roasting treatments. The preliminary samples were then subjected to sequential roasting as follows: the first roasting at 110-115 °C for 1 h (defined as the initial roasting stage, RGZS); the second roasting at 110-115 °C for 16 h (defined as the first repeated roasting stage, RG1O); the third roasting at 110-115 °C for 16 h (defined as the second repeated roasting stage, RG2O); and the final roasting at 110-115°C for 16 h (defined as the third repeated roasting stage, RG3O). Roasting was performed at intervals of approximately 45 days, with samples collected after each stage.

After each roasting treatment, samples were cooled to room temperature, sealed, and stored under dark conditions until subsequent analysis. The detailed information regarding the roasting treatments, sampling points, and storage conditions is summarized in **Supplementary Table 1**. All tea samples were stored under dark and room-temperature conditions for subsequent analyses. Detailed information on all reagents and chemicals used in this study is provided in the **Supplementary Materials**.

### 2.2 Sensory evaluation analysis

#### 2.2.1 Sensory assessment

Sensory evaluation of the four Rougui tea samples was conducted following the oolong tea evaluation protocol described in the Chinese National Standard GB/T 23776-2018. For each sample, 5 g of tea was placed in a 110 mL covered bowl, and boiling purified water was added. The infusions were brewed for 2, 3, and 5 min, sequentially filtered into evaluation cups, and assessed for aroma and taste. The sensory panel consisted of seven experienced evaluators (four females and three males, aged 26–45 years), all holding senior tea tasting certificates with 5–20 years of professional experience in tea sensory evaluation. Before the formal evaluation, all panelists received standardized training on sensory descriptors and evaluation procedures to ensure consistency among assessments. All samples were randomly coded, and panelists evaluated appearance, aroma, liquor color, taste, and infused leaf quality according to the terminology defined in GB/T 14487-2017. The overall sensory score was calculated based on a 100-point scale with the following assigned weights: appearance (20%), liquor color (5%), aroma (30%), taste (35%), and infused leaves (10%). The final score for each sample was calculated as the mean of the panelists’ individual comprehensive ratings.

#### 2.2.2 Sensory training and QDA

Prior to formal evaluation, the panelists underwent a one-month sensory training program using a previously established flavor description system and Rougui samples not included in the experiment. Training focused on aroma recognition, description, and consensus in perception, until panelists were able to consistently apply the sensory rating scale. Aroma attributes included floral, cinnamon, sweet, roasted, green, and sour notes. Taste attributes included thickness, intensity, sweet aftertaste, umami, briskness, bitterness, astringency, smoothness, and sourness. Tea infusion and scoring procedures followed the method described above, with each attribute evaluated on a 10-point scale (0 = not perceptible, 2 = slightly perceptible, 4 = moderately perceptible, 6 = distinct, 8 = strong, 10 = very strong). Quantitative descriptive analysis (QDA) was then applied to systematically quantify and compare the sensory profiles across different samples. Evaluations were conducted independently, with each panelist performing three replicates per sample and five independent assessments per session. The final score for each attribute was calculated as the average of the seven panelists’ ratings.

#### 2.2.3 Electronic sensory analysis

Electronic tongue: An ASTREE LS48 electronic tongue system (Alpha-MOS, France) equipped with a sensor array of seven highly sensitive sensors (AHS, PKS, CTS, NMS, CPS, ANS, and SCS) was used for taste analysis. For each measurement, a tea-to-water ratio of 1:22 was used, and the filtered tea infusion was used as the test sample. Each sample was analyzed in eight replicates, with a data acquisition time of 120 s and a sampling interval of 1 s. The stable data from the last three replicates were selected for statistical modeling.

IRIS electronic eye: An IRIS electronic eye system (Alpha-MOS, France) was employed to evaluate the appearance of dry tea leaves, infused leaves, and tea liquor. Thirty dry or infused leaves were randomly selected and evenly arranged on a white imaging tray, or 25 mL of tea liquor was transferred into a dedicated cuvette for analysis. A dual-axis top and bottom illumination system was used during imaging to ensure uniform lighting, thereby improving image consistency and analytical accuracy.

An electronic nose system (PEN3, Airsense, Germany) equipped with ten metal oxide semiconductor (MOS) gas sensors was used to assess aroma characteristics. The sensor array included W1C (aromatic compounds and benzene), W5S (nitrogen oxides), W3C (ammonia and aromatic compounds), W6S (hydrogen), W5C (short-chain alkanes and aromatics), W1S (methyl compounds), W1W (inorganic sulfides and terpenes), W2S (alcohols, ethers, aldehydes, and ketones), W2W (aromatic compounds and organic sulfides), and W3S (long-chain alkanes), with detailed sensor characteristics provided in **Supplementary Table 2**. After preheating and purging for 30 min, 1.0 g of tea sample was placed in a 100 mL beaker sealed with double-layer plastic film, equilibrated at room temperature for 30 min, and then heated at 80°C for 5 min. Data acquisition was performed for 90 s at an injection flow rate of 400 mL/min, followed by a 120 s cleaning cycle. Each sample was analyzed in triplicate.

### 2.3 GC-MS analysis of samples

#### 2.3.1 Sample preparation and GC-MS analysis

A total of 500 mg of tea powder was placed into a headspace vial, followed by the addition of 2 mL saturated NaCl solution and 20 μL of internal standard solution (3-Hexanone-2,2,4,4-*d*_4_, 10 μg/mL). The mixture was equilibrated under constant 80 °C shaking for 5 min. Headspace extraction was performed using a 120 μm DVB/CWR/PDMS fiber needle for 15 min, followed by desorption in a splitless inlet at 250 °C for 5 min prior to analysis. Before sampling, the SPME fiber was conditioned in a fiber conditioning station at 250 °C for 5 min to remove residual impurities.

Chromatographic separation was performed using an Agilent 8890 GC coupled with a 7000D MS system, equipped with a DB-5MS capillary column (30 m × 0.25 mm × 0.25 μm; Agilent J&W Scientific, Folsom, CA, USA). The injection port was operated in splitless mode at 250 °C, with high-purity helium (≥99.999%) as the carrier gas at a constant flow of 1.2 mL/min. The oven temperature program was set as follows: initially 40 °C held for 3.5 min, ramped at 10 °C/min to 100 °C, then at 7 °C/min to 180 °C, and finally at 25 °C/min to 280 °C, held for 5 min. Mass spectrometry was performed using an electron impact (EI) ion source at 70 eV. The quadrupole, ion source, and transfer line temperatures were set to 230 °C, 150 °C, and 280 °C, respectively. Detection was conducted in selected ion monitoring (SIM) mode for precise qualitative and quantitative analysis. Each sample was analyzed in triplicate.

#### 2.3.2 Identification, quantification, and OAV calculation of volatile metabolites

A self-established database was constructed based on multiple species, literature data, selected authentic standards, and retention indices, containing verified retention times (RT) and qualitative and quantitative ions. Selected ion monitoring (SIM) was used for precise detection, with one quantitative ion and two to three qualitative ions chosen for each compound. All ions to be monitored in a given set were detected sequentially according to their elution order and within defined time segments. A compound was identified when its retention time matched the standard reference and all selected ions were present in the sample mass spectrum after background subtraction (Yuan et al., 2021). Quantitative ions were used for peak integration and calibration to improve quantification accuracy. Relative concentrations of volatile compounds (μg/g) were calculated using the internal standard method, based on the ratio of the target peak area to the internal standard peak area. Odor activity values (OAVs) were defined as the ratio of the compound concentration to its sensory threshold in water, as reported in the literature, and were calculated using Equation 1:

(1) $$OAV=\frac{C_{x}}{OT_{X}}$$

In the equation: *C_x_*, concentration of volatile compound x (µg/g); *OT_X_*, odor threshold of volatile compound x in water (µg/g).

### 2.4 Determination of non-volatile metabolites

#### 2.4.1 Catechins and alkaloids

Catechins, caffeine, and theobromine were quantified using an Ultimate 3000 UHPLC-DAD system (Thermo Fisher Technologies (China) Co., LTD.). For extraction, 0.2 g of tea powder was extracted twice with 5 mL of 70% methanol at 70 °C for 10 min each, with intermittent shaking. The extracts were centrifuged at 3500 rpm for 10 min, combined, and brought to a final volume of 10 mL. After filtration through a 0.45 μm membrane, the sample was diluted fivefold with stabilizing solution containing EDTA-Na_2_, ascorbic acid, and acetonitrile. Separation was performed on a C18 reversed-phase column (5 μm, 4.6 × 250 mm) using a gradient of 0.2% formic acid (A) and methanol (B) at a flow rate of 1.0 mL/min, with column temperature maintained at 40 °C. Detection wavelengths were set at 278 nm and 280 nm.

#### 2.4.2 Thearubigins and theabrownins

The contents of thearubigins and theabrownins were determined using a UV–visible spectrophotometer, with sequential extractions performed using n-butanol and ethyl acetate. Tea samples were first extracted with boiling water and then partitioned into three phases: (A) oxalic acid/ethanol solution, (B) the aqueous phase after n-butanol extraction, and (C) the organic phase obtained by ethyl acetate/sodium bicarbonate extraction. Absorbance values were measured at 380 nm (E_A_, E_B_, E_C_) and used for calculations using Equations 2 and 3:

(2) $$X_{TR}=\frac{16.994\times E_{B}}{m\times \omega }\times 100\%$$

(3) $$X_{TB}=\frac{16.994\times \left(E_{A}-\frac{E_{C}}{2}-E_{B}\right)}{m\times \omega }\times 100\%$$

where E_A_, E_B_, and E_C_ are the corresponding absorbance values, m is sample mass, and ω is the dry matter coefficient.

#### 2.4.3 Detection of amino acid and nucleotide components

Amino acids were analyzed using an LC-20ADXR (Shimadzu, Kyoto, Japan) coupled with a Sciex 5500+ triple quadrupole mass spectrometer (Sciex, Singapore). 100 mg of tea powder was extracted with 10 mL preheated methanol at 70 °C for 20 min using ultrasonication. After centrifugation and filtration, 10 μL internal standard was added, and 2 μL was injected. Separation was performed on an Acquity UPLC BEH Amide column (1.7 μm, 2.1 × 100 mm) with mobile phases A (90% acetonitrile in water) and B (50% acetonitrile in water), both containing ammonium formate and formic acid buffer. A linear gradient to 70% B was applied over 12 min at a flow rate of 0.3 mL/min. Mass spectrometry was performed using a Turbo V™ ion source in SRM mode, with rapid switching between positive and negative ionization, ion source temperature of 550 °C, and curtain gas at 35 psig. Data were processed using Sciex OS software, and quantification was based on isotopically labeled internal standards.

#### 2.4.4 Organic acids and soluble sugars

Sample preparation was identical to section 2.4.3. After adding 5 μL internal standard, 20 μL 3-nitrophenylhydrazine and 20 μL EDC reagent were added for derivatization at 30 °C for 60 min. The derivatized samples were reconstituted and separated on an Acquity UPLC HSS C18 column using 0.01% formic acid in water (A) and acetonitrile (B) with a 12 min gradient from 0% to 95% B. MS parameters and quantification followed the amino acid protocol.

#### 2.4.5 Determination of flavonol glycosides, phenolic acids

100 mg of each sample was placed into a 15 mL centrifuge tube with 10 mL of preheated methanol. The mixture was ultrasonicated in a 70 °C water bath for 20 min, then cooled to room temperature and centrifuged at 3,500 rpm for 10 min. The supernatant was filtered through a 0.22 μm membrane for analysis. Metabolites were separated on an Acquity UPLC HSS C18 column (1.8 μm, 2.1 × 100 mm) using a Thermo Ultimate 3000 UHPLC (Thermo Scientific, USA) system with a binary mobile phase of 0.01% formic acid in water (A) and acetonitrile (B). The flow rate was 0.3 mL/min, and a linear gradient from 0% to 90% B was applied over 12 min. A TSQ Endura MD Plus mass spectrometer with heated electrospray ionization (ESI) was used in scheduled SRM mode. Mass spectrometry (Thermo Scientific, USA) parameters were: +3.5 kV (positive)/–3.0 kV (negative) spray voltage, 40 Arb sheath gas, 15 Arb auxiliary gas, 2.0 mTorr collision gas, and 350 °C heater temperature. Raw data were processed with TraceFinder software (Thermo Scientific, USA), and metabolites were quantified using external standard calibration. Detailed information regarding the reagents and chemicals utilized for the determination of non-volatile metabolites is provided in the **Supplementary Material**.

#### 2.4.6 Taste contribution of non-volatile components

To quantify the sensory contribution of core non-volatile metabolites to the taste of Rougui tea infusion, the dose-over-threshold (DOT) index was introduced. DOT values were defined as the ratio of the measured concentration of a target compound to its sensory threshold, as shown in Equation 4:

(4) $$DOT_{i}=\frac{C_{i}}{T_{i}}$$

where *Ci* represents the concentration of non-volatile metabolite i in the tea infusion (μg/mL), and *Ti* is its taste threshold in water or simulated tea infusion (μg/mL). Sensory thresholds for core metabolites, such as gallic acid (GA), and gallocatechin gallate (GCG), were referenced from previous literature (Yu et al., 2014; Zhu et al., 2021). Compounds with DOT > 1 were considered to significantly contribute positively or negatively to tea taste, with the DOT value reflecting the strength of the contribution.

### 2.5 Determination of proteins and pectin components

Soluble protein content was measured using a BCA Protein Assay Kit (UPLC-MS-4502, Shanghai Liquid Mass Tech Co., Ltd.). Approximately 0.1 g of tea powder was extracted with 1 mL PBS buffer and centrifuged at 12,000 rpm for 10 min at 4 °C. Twenty microliters of supernatant was mixed with 200 μL BCA working solution in a 96-well plate at a 50:1 ratio, incubated at 37 °C for 30 min in the dark, and absorbance was measured at 562 nm. Pectin components, including water-soluble pectin (WSP) and protopectin, were determined using commercial kits (UPLC-MS-4127 and UPLC-MS-4125, Shanghai Liquid Mass Tech). Total pectin content was estimated as the sum of water-soluble pectin and protopectin according to the following equation: Total pectin content = Protopectin content + Water-soluble pectin content. Samples were first converted to alcohol-insoluble residue (AIR) by extracting 0.1 g of tea sample with 1 mL of 80% ethanol at 95 °C for 20 min, followed by centrifugation at 4000 g for 10 min. The residues were washed with acetone, then extracted with distilled water for WSP determination and with acid solution in a boiling water bath for protopectin determination. Extracts reacted with carbazole reagent under acidic conditions to produce purple-colored compounds, and pectin content was calculated based on absorbance at 530 nm using a galacturonic acid standard curve.

### 2.6 Molecular docking

Molecular docking was performed following previously reported methods (Liu et al., 2022; Liang et al., 2024), integrating metabolomic and sensory data. Gallic acid and gallocatechin gallate were selected as representative taste-related compounds for docking with the calcium-sensing receptor (CaSR, PDB ID: 7B6W), which has been reported to be associated with kokumi-related taste perception. Volatile aroma compounds, including 1-nonen-3-one, (*E*, *E*)-2,4-heptadienal, hotrienol (dehydro-linalool), (2-methylpropyl) pyrazine, 5-ethyl-2-methylpyridine, and indole, were docked with the broadly tuned human olfactory receptor OR1A1, which was selected as a representative olfactory receptor for predicting potential ligand–receptor interactions. Receptor structures were obtained from UniProt (https://www.uniprot.org/), and ligand structures from PubChem (https://pubchem.ncbi.nlm.nih.gov/). Preprocessing and docking were performed using CB-Dock 2. The predicted binding energies (kcal/mol) were used as indicators of potential ligand–receptor interaction strength rather than direct evidence of receptor activation. Docking results were visualized with Discovery Studio 4.5 Client (Dassault Systèmes, France).

### 2.7 Statistical analysis

All experiments were conducted in triplicate, and results are expressed as mean ± standard deviation. One-way ANOVA and Tukey’s HSD multiple comparison tests (*p* < 0.05) were performed using SPSS 26.0. Principal component analysis (PCA) and orthogonal partial least squares discriminant analysis (OPLS-DA) were performed on the Metware Cloud Platform, with variable importance in projection (VIP) > 1.0 used to identify key markers. Data visualization was performed using Origin 2021 and GraphPad Prism 9. Electronic tongue and electronic eye data were processed and plotted using AlphaSoft software provided by the instrument manufacturer.

## 3 Results and discussion

### 3.1 Sensory evaluation of samples at different roasting stages

**Figure 1:**
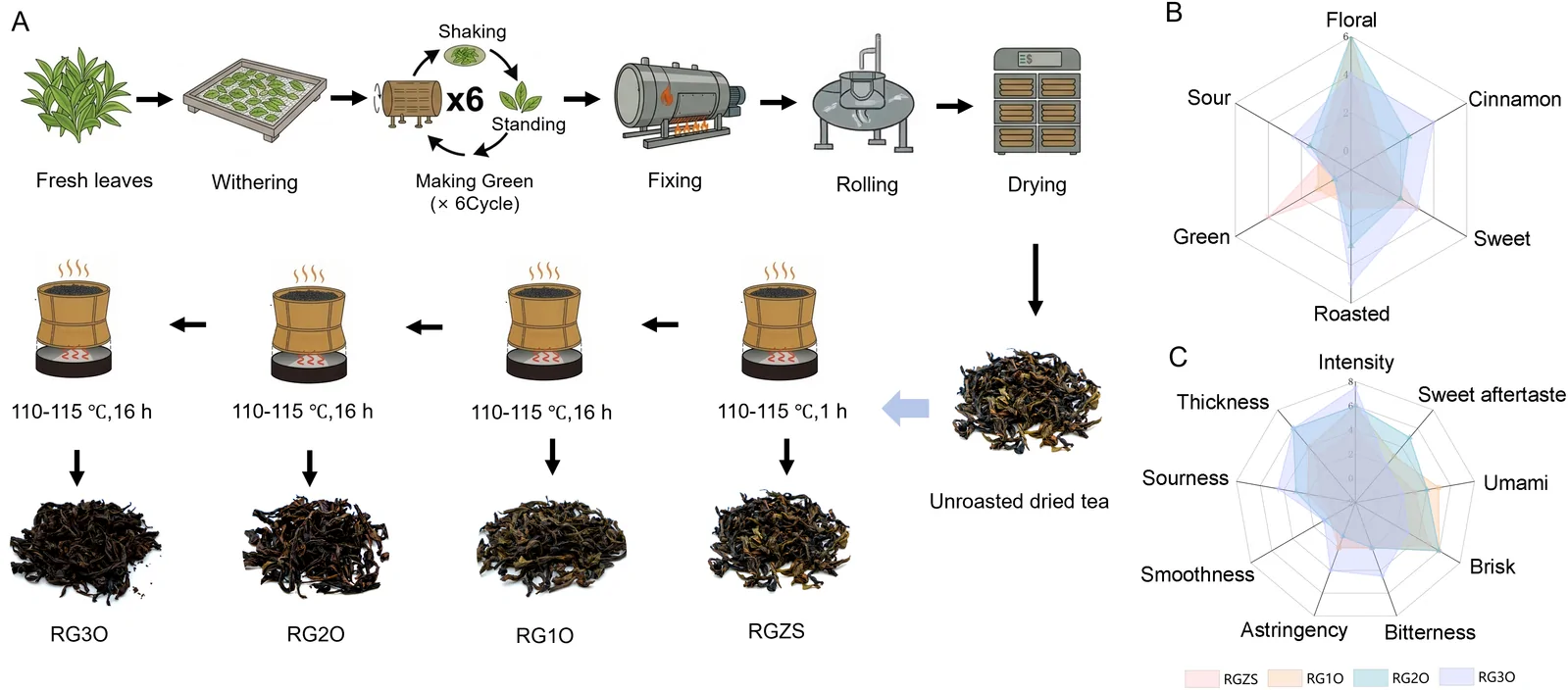
Processing workflow of Wuyi Rougui tea and sensory characteristics of samples under different roasting treatments. **(A)** Schematic diagram of the processing workflow from fresh leaves to dried tea and the preparation of Wuyi Rougui tea samples under different roasting treatments. **(B, C)** Quantitative descriptive analysis of aroma and taste attributes, respectively, in Wuyi Rougui tea samples under different roasting treatments.

Sensory evaluation is essential for capturing the integrated flavor evolution of tea during thermal processing (Zeng et al., 2020). Therefore, the dynamic sensory profiles of Rougui across four roasting stages were systematically assessed in this study (**Table 1**). **Figure 1A** shows the complete processing workflow from fresh leaves to unroasted dried tea, followed by different roasting treatments. As roasting progressed, the color of the dried tea changed from yellow-green to dark brown. The aroma QDA results showed that green and floral attributes were predominant at the RGZS stage. At RG1O, the floral and sweet attributes increased significantly. During the second roasting stage (RG2O), the cinnamon attribute increased markedly, while the floral attribute reached its maximum, indicating that the balance of these two aroma components represents the characteristic sensory profile of Rougui. At RG3O, the roasted attribute reached the highest score, reflecting the predominance of roasted aroma in the final stage (**Figure 1B**). Overall, aroma evolved from fresh and floral notes toward dominant roasted characteristics with increasing roasting intensity.

**Table 1:** Sensory evaluation of Wuyi Rougui at different roasting stages.

Sample	Appearance					Inner quality								Total score
	Shape	Color	Integrity	Clarity	Score (20%)	Liquor color	Score (5%)	Aroma	Score (30%)	Taste	Score (35%)	Infused Leaves	Score (10%)	
RGZS	Fairly tightly twisted	Dark brown with sand-green	Even and uniform	Clear	18.0	Light orange-yellow, bright	4.6	Persistent floral-fruity aroma	26.4	Mellow and refreshing	30.5	Soft, even, distinct red edges	8.7	88.2
RG1O	Tightly twisted	Dark brown	Even and uniform	Clear	18.2	Orange-yellow, bright	4.6	Floral-fruity, moderately intense	27.0	Mellow, thick, and brisk; notable cultivar attributes	31.5	Soft, even, with red edges and residual fragrance	9.0	90.3
RG2O	Tightly twisted	Dark brown	Even and uniform	Clear	18.2	Light orange-red, bright	4.6	Intense floral-fruity aroma with cinnamon notes	28.2	Mellow, thick, and sweet-brisk; distinct cultivar attributes	32.6	Fairly soft, even, red edges, notable residual fragrance	9.3	92.9
RG3O	Tightly twisted	Dark and lustrous	Even and uniform	Clear	17.8	Orange-red, fairly bright	4.5	Persistent floral-fruity aroma; distinct roasted and cinnamon notes	26.1	Strong, mellow, and moderately thick with subtle acidity; notable cultivar attributes	30.8	Moderately soft, even, with residual fragrance	8.6	87.8

Taste QDA results demonstrated significant changes in all attributes along the roasting gradient. Liquor thickness and intensity increased progressively with roasting and peaked at RG3O, indicating that thermal treatment enhances the taste structure. Sweet aftertaste reached its maximum at RG2O, while briskness and umami were highest at RG1O. Notably, astringency was lowest at RG2O but increased at RG3O, likely due to prolonged roasting promoting the accumulation of tannins and elevating the perception of bitterness. Integrated analysis indicated that RG2O represents an optimal sensory balance for Rougui. At this stage, the grassy notes present in preliminary tea were effectively removed, while the characteristic cinnamon and floral aromas were maximally preserved and enhanced. Liquor thickness, sweet aftertaste, and smoothness all reached high levels, resulting in optimal sensory coordination. Although RG3O further increased thickness and intensity, bitterness and sourness also rose, potentially negatively affecting overall sensory acceptance. This sensory reconstruction is consistent with previous studies, primarily attributable to the loss of low-boiling volatile compounds and the contribution of Maillard reactions to the enhancement of liquor thickness and aroma complexity (Wang et al., 2022; Yang et al., 2022). Such thermally driven optimization of sensory coordination not only provides a distinctive flavor differentiation for grading Rougui (Ye et al., 2025) but also aligns with the quality evolution patterns observed in other oolong teas, such as Tieguanyin, during roasting (Cao et al., 2021; Song et al., 2023).

### 3.2 Electronic sensory analysis

**Figure 2:**
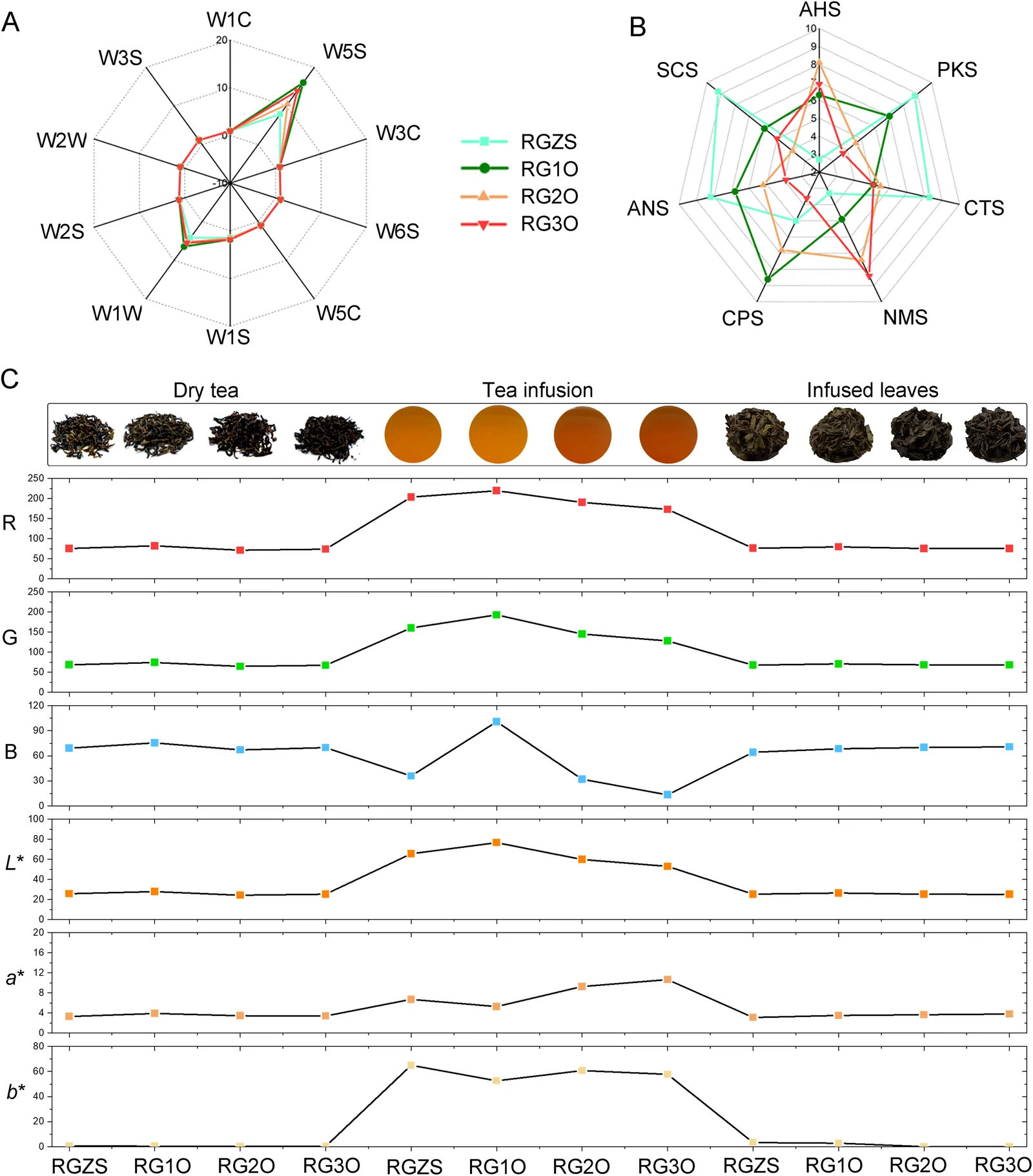
Electronic sensory characteristics of Wuyi Rougui tea samples under different roasting treatments. **(A)** Radar plot of electronic nose responses from ten metal oxide semiconductor (MOS) sensors. **(B)** Radar plot of seven taste attributes measured by the electronic tongue. **(C)** RGB values and CIELAB color parameters (L^*^, a^*^, and b^*^) of samples under different roasting treatments. The electronic eye images from left to right show dry tea, tea infusion, and infused leaves, respectively.

Radar plots were constructed based on the response intensities of the electronic nose sensor array across different roasting stages of Rougui (**Figure 2A**). Sensor responses indicated that all samples exhibited the strongest response on the W5S sensor, with RG1O showing the highest response, followed by RG3O, RG2O, and RGZS. This suggests that nitrogen oxides were the dominant aroma contributors during roasting, while W5S also reflected the diversity of volatile compounds in the tea samples. The second strongest responses were observed on the W1W sensor, consistent with W5S trends, indicating its sensitivity to sulfur-containing volatiles. Notably, RG3O exhibited the highest response on the W1S sensor, suggesting that high-temperature roasting promoted the formation of methylated heterocyclic compounds via Maillard and caramelization reactions, providing the chemical basis for the “roasted aroma” characteristic of Rougui. Responses from other sensors showed minor variation, indicating that the corresponding volatile compounds remained relatively stable or at low concentrations, contributing minimally to the primary aroma profile.

Electronic tongue measurements were used to analyze taste changes of Rougui across roasting stages (**Figure 2B**). RGZS samples showed the highest response on the SCS sensor, followed by PKS, ANS, and CTS. With increasing roasting intensity, responses of these sensors gradually decreased, whereas AHS, NMS, and CPS responses increased, indicating that prolonged roasting reduced bitterness while enhancing umami. RG2O samples exhibited the lowest SCS response, suggesting that the second roasting stage represents an optimal point for reducing bitterness and astringency, likely related to the thermal degradation of ester-type catechins and targeted polymerization of polyphenols. AHS responses increased significantly with roasting and peaked at RG2O, consistent with the trend observed in sourness ratings, likely due to stepwise accumulation of organic acids such as gallic acid. However, NMS responses increased steadily with roasting, a trend diverging from the QDA results for freshness, which initially increased and then decreased. This discrepancy suggests that the NMS sensor captures complex responses to nitrogen-containing compounds generated during roasting, rather than traditional freshness alone. This may be attributed to the strong umami-enhancing effects of pyridine derivatives formed via the Maillard reaction or the contribution of pyroglutamate to a more complex savory-acidic profile, leading to sustained enhancement of NMS signals (Feng et al., 2024). Although NMS responds to pyridine derivatives, its correlation with human perception of umami requires further validation through sensory experiments.

Finally, an electronic eye system was used to analyze the effects of different roasting treatments on the color characteristics of dry tea, tea infusions, and infused leaves of Wuyi Rougui tea (**Figure 2C**). For dry tea and tea infusions, the RGB values and L^*^ and a^*^ values of RG1O were higher than those of the other treatments, indicating that the first roasting treatment increased sample brightness. For dry tea, the RGB and L^*^ values decreased after the first roasting treatment, although the changes from RG2O to RG3O were not strictly monotonic. Meanwhile, the b^*^ value decreased continuously from RGZS to RG3O, indicating a progressive reduction in yellowness. These changes suggest that roasting promoted the transition of dry tea from a relatively bright yellow-green appearance toward darker brown tones. In contrast, tea infusions were more sensitive to roasting treatments. The first roasting treatment markedly increased the brightness of tea infusions, whereas continued roasting gradually deepened the liquor color, with continuous decreases in RGB and L^*^ values. Meanwhile, the a^*^ value gradually increased from RG1O to RG3O, indicating enhanced redness. The b^*^ value fluctuated among the roasted samples rather than decreasing continuously; however, it remained lower in all roasted samples than in RGZS, suggesting an overall reduction in yellowness after roasting. Overall, roasting promoted the color transition of tea infusions from bright orange-yellow to reddish-brown.

For infused leaves, the R, G, and L^*^ values showed only minor fluctuations with increasing roasting intensity, indicating that their overall brightness remained relatively stable. In contrast to the original interpretation, the B value increased moderately rather than decreasing. The a^*^ value increased progressively from RGZS to RG3O, whereas the b^*^ value decreased markedly and approached zero. These changes indicate a slight enhancement of redness and a pronounced reduction in yellowness in the infused leaves. Overall, the first roasting treatment increased the brightness of dry tea and tea infusions, while further roasting reduced brightness and promoted redness in tea infusions. In contrast, the color of infused leaves changed relatively little, although their yellowness decreased continuously and their redness increased slightly with roasting intensity.

### 3.3 Characterization of volatile metabolites in Rougui at different roasting stages

#### 3.3.1 Composition of volatile metabolites and discrimination analysis

**Figure 3:**
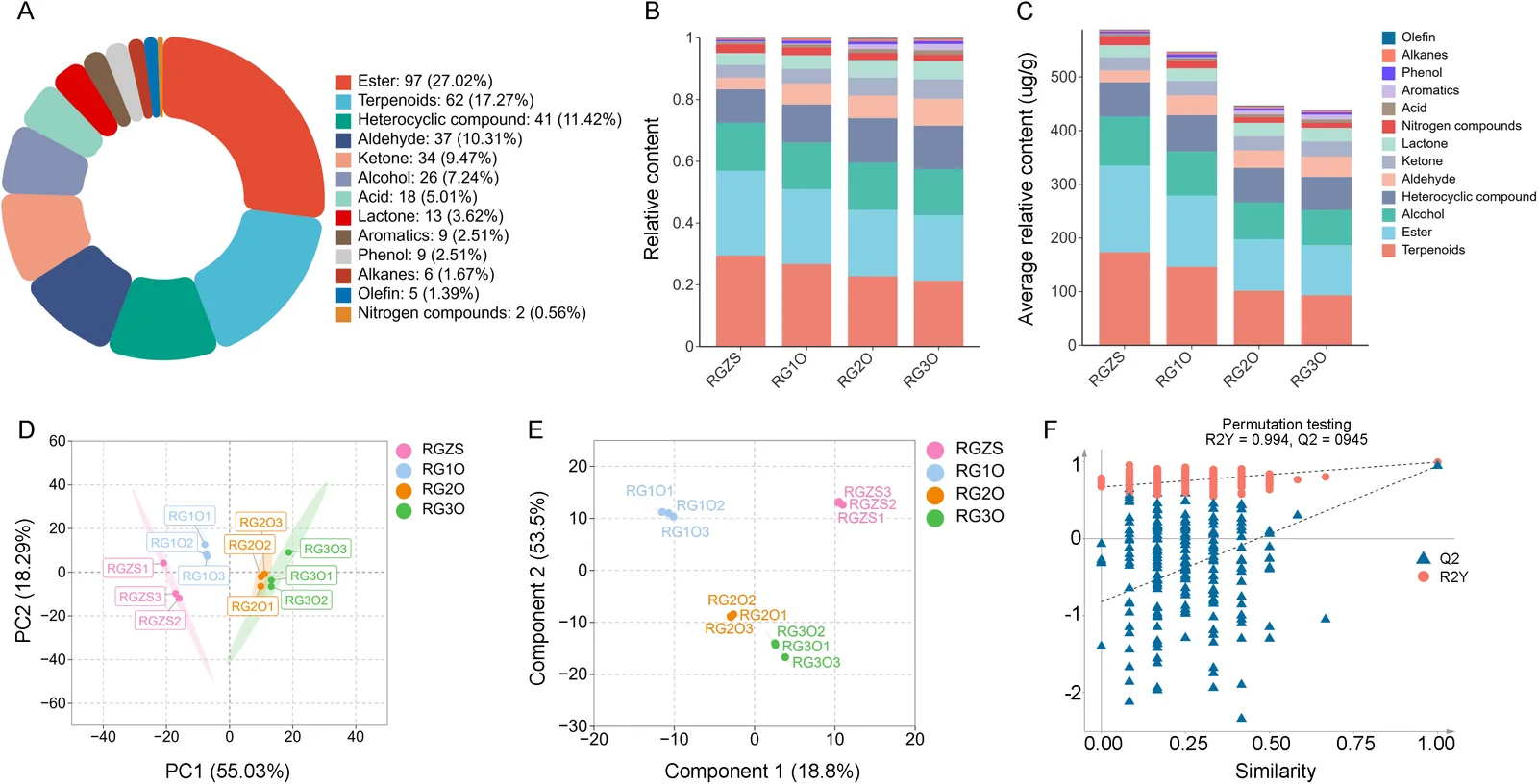
Multivariate statistical analysis of Volatile metabolites in Wuyi Rougui. **(A)** Doughnut chart of VM classifications. **(B)** Stacked bar chart of the relative percentages of VMs. **(C)** Stacked bar chart of average volatile metabolites contents. **(D)** PCA score plot. **(E)** OPLS-DA score plot. **(F)** Permutation test results for the OPLS-DA model.

Volatile metabolites are important components of tea aroma and are affected by roasting-associated chemical transformations, including glycoside hydrolysis, lipid oxidation, and Maillard-related reactions (Yang et al., 2023). Wuyi Rougui, a core cultivar of Wuyi rock tea, is characterized by a rich floral and fruity aroma and a uniquely sharp, persistent cinnamon-like fragrance (Liang et al., 2024). In this study, the HS-SPME-GC-MS system was employed to profile volatile metabolites across different roasting stages, aiming to characterize the dynamic changes in volatile composition associated with the development of roasted aroma characteristics. Results indicated that roasted Rougui exhibited a rich and diverse aroma profile. A total of 362 volatile compounds were identified, encompassing 14 categories, including esters, terpenes, heterocyclic compounds, and alcohols (**Figure 3A**). Among these, esters were the most abundant (97 compounds, 27.0%), followed by terpenes (62 compounds, 17.3%) and heterocyclic compounds (41 compounds, 11.4%). These three categories together accounted for more than half of the total volatile content, representing the main composition framework of Rougui aroma.

Quantitative analysis of volatiles revealed that terpenes, esters, heterocyclic compounds, and alcohols together accounted for 71.28%–83.40%% of the total volatile content, with terpenes being the highest, followed by esters and alcohols (Yan et al., 2025). As roasting intensity increased, the levels of these major volatiles showed a decreasing trend, with total volatile content decreasing by approximately 25.08% across roasting stages (**Figures 3B, C**), suggesting that roasting was associated with substantial changes in the volatile composition of Rougui. This feature is consistent with dynamic metabolomic studies of the entire Rougui processing workflow (Liu et al., 2022) and has also been observed in other oolong teas, such as Shuixian tea (Duan et al., 2025; Zheng et al., 2025), as well as in specialty floral oolong teas (Zhang et al., 2025). Moreover, large-scale comparative GC-MS analyses between Rougui and Shuixian tea confirmed that heat-associated changes in aroma profiles may contribute to differences in aromatic complexity among Wuyi tea cultivars (Wang et al., 2024). Principal component analysis (PCA) based on quantitative volatile data revealed a clear separation of samples from different roasting stages in the scatter plot (**Figure 3D**). The first two principal components explained 55.03% (PC1) and 18.29% (PC2) of the variance, respectively, with a cumulative contribution of 73.32%. Samples were clearly distinguished along the PC1 axis, indicating that roasting intensity is the primary factor driving the differentiation of aroma metabolite profiles. Further orthogonal partial least squares discriminant analysis (OPLS-DA) effectively separated the sample groups (**Figure 3E**). Permutation tests yielded R²Y = 0.994, Q² = 0.945, with a positive regression slope and a significantly negative Q² intercept, suggesting good model fitting and predictive performance for distinguishing volatile metabolite profiles among different roasting stages. (**Figure 3F**). In the OPLS-DA score plot, RGZS and RG1O were positioned on opposite sides of the upper region, showing clear separation along component 1 (18.8%), reflecting significant differences in major metabolic variations between these two groups. RG1O, RG2O, and RG3O were relatively distant from each other, indicating that volatile metabolite profiles underwent continuous remodeling during repeated roasting, with the characteristic roasted aroma-associated metabolites becoming more pronounced at later roasting stages, consistent with previous characterization of aroma activity in differently roasted Rougui teas (Yang et al., 2022). Using a threshold of VIP > 1 and *p<* 0.05, a total of 76 differential volatile compounds were identified.

#### 3.3.2 Differential volatile compounds and OAV analysis

**Figure 4:**
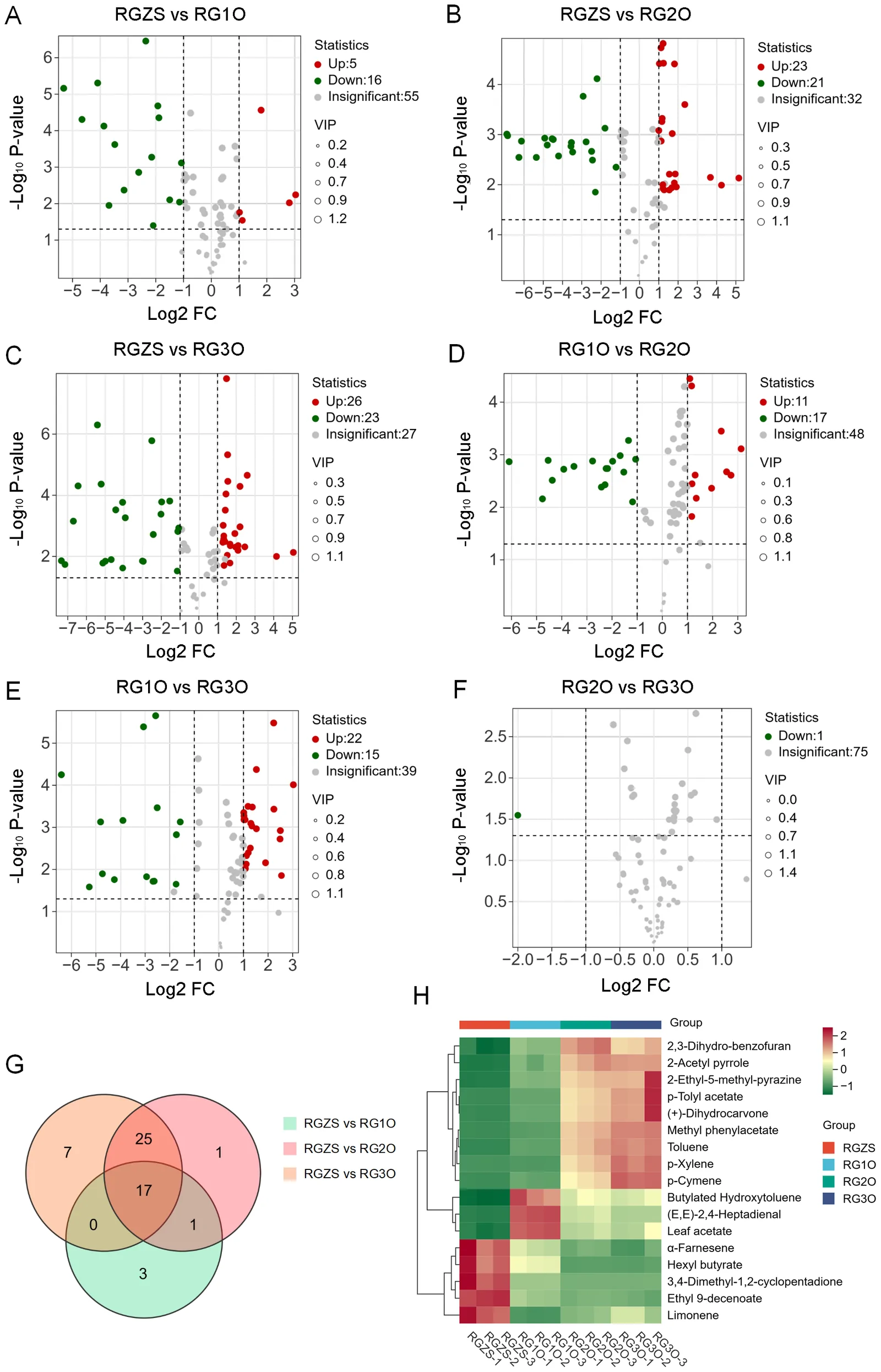
Differential analysis of volatile compounds in Wuyi Rougui. **(A–F)** Volcano plots showing pairwise comparisons among samples from different roasting stages. Differential compounds were identified based on p< 0.05 and log_2_ fold change thresholds. **(G)** Venn diagram of RG1O, RG2O, and RG3O compared with RGZS. **(H)** Clustered heat map of shared compounds in the RG1O, RG2O, and RG3O versus RGZS comparison groups.

Based on *p* < 0.05 and log_2_ fold change (Log_2_FC) thresholds, the 76 differential volatile compounds (**Supplementary Table 3**) were further analyzed to assess significant changes across comparisons. Results showed that, relative to RGZS samples, the number of significantly up- and down-regulated compounds increased with the number of roasting cycles (**Figures 4A–C**). The comparison between RGZS and RG3O exhibited the highest number of both up- and down-regulated compounds. Notably, significantly upregulated compounds included dihydrocarvone, benzaldehyde, 2-ethyl-5-methyl-pyrazine, and *p*-xylene, whereas significantly downregulated compounds included 3,4-dimethyl-1,2-cyclopentadione, hexyl butyrate, (*E*)-β-ionone, geraniol, and ethyl 9-decenoate. These results suggest that prolonged roasting was associated with substantial remodeling of the volatile metabolite profile, including increased accumulation of some Maillard-related heterocyclic compounds and decreased levels of several heat-sensitive esters and terpenoids. Among different roasting stage comparisons, RG2O vs RG3O showed the smallest variation, with only myrac aldehyde significantly downregulated, suggesting that the overall changes in volatile profiles became relatively limited between these two later roasting stages (**Figures 4D–F**). A Venn diagram comparing three groups of samples against RGZS revealed that RGZS vs RG3O contained more unique compounds than other comparisons, indicating greater differences in volatile composition between the initial and final roasting stages. Seventeen compounds were common across all three comparisons, representing the most stable volatiles that significantly changed across all roasting stages (**Figure 4G**). Hierarchical clustering of these 17 compounds categorized them into three groups (**Figure 4H**). The first cluster comprised *α*-farnesene, hexyl butyrate, 3,4-dimethyl-1,2-cyclopentadione, ethyl 9-decenoate, and limonene, all of which were most abundant in RGZS and generally decreased after roasting. The second cluster included (*E, E*)-2,4-heptadienal, leaf acetate, and butylated hydroxytoluene, which increased sharply and reached their highest levels in RG1O, followed by a decline during subsequent roasting stages. The third cluster consisted of 2,3-dihydrobenzofuran, 2-acetyl pyrrole, 2-ethyl-5-methyl-pyrazine, *p*-tolyl acetate, (+)-dihydrocarvone, methyl phenylacetate, toluene, *p*-xylene, and *p*-cymene. These compounds were present at relatively low levels in RGZS but showed increased abundances during roasting, with generally higher abundances in RG2O and RG3O. Notably, 2,3-dihydrobenzofuran reached its maximum level in RG2O, whereas (+)-dihydrocarvone, 2-ethyl-5-methyl-pyrazine, methyl phenylacetate, *p*-tolyl acetate, toluene, *p*-xylene, and *p*-cymene showed their highest levels in RG3O.

The aroma evolution of Rougui across roasting stages showed a stage-dependent shift from fresh, citrus–herbal, and floral–fruity attributes toward increasingly roasted and nutty characteristics, rather than a strictly monotonic attenuation of all green volatiles (**Supplementary Table 4**). At the starting RGZS stage, relatively high OAVs were observed for limonene (191.41 ± 22.92), 3,4-dimethyl-1,2-cyclopentadione (90.74 ± 13.27), *α*-farnesene (22.88 ± 3.15), and hexyl butyrate (16.58 ± 2.77), which may have contributed to the perception of citrus–herbal, green, floral–fruity, and caramel-like aroma attributes. Some of these volatiles may originate from the release or transformation of aroma precursors during tea processing (Chen et al., 2025b). Limonene decreased sharply at RG1O (23.48 ± 1.74) and then partially recovered at RG2O and RG3O (53.16 ± 5.40 and 77.97 ± 14.71, respectively), whereas *α*-farnesene and hexyl butyrate declined progressively across the roasting stages. These patterns suggest selective changes and remodeling of the fresh aroma-related volatile profile rather than a uniform loss of all fresh or green volatiles.

At the first roasting stage, the OAV of (*E, E*)-2,4-heptadienal increased from an undetectable level at RGZS to 213.97 ± 4.80 at RG1O, before decreasing to 100.26 ± 6.62 at RG2O and 83.38 ± 0.82 at RG3O. This transient peak suggests rapid formation during early roasting followed by further degradation or conversion during prolonged heating. Based on its odor characteristics, this compound may be associated with fatty, green, oily, and cinnamon-like aroma attributes and may represent a potential candidate compound reflecting the transition between RGZS and the first roasting stage.

During RG2O and RG3O, Maillard reactions and other thermally induced transformations were likely associated with increased levels of several roasted-aroma-related compounds. Previous studies have shown that theanine and other amino acids can participate in deamination and Strecker-related reactions, leading to the formation of volatile heterocyclic and pyrazine compounds (Guo et al., 2018; Li et al., 2022). Isotope-labeled leaf-based models have further confirmed roasting-induced formation pathways of alkyl pyrazines and furan derivatives (Chen et al., 2025a), while CAMOLA-based carbon-module labeling has enabled precise tracking of furan and pyrrole formation in Maillard systems (Gao et al., 2025). In addition to amino acids and reducing sugars, catechins and other tea polyphenols may also participate in the formation and transformation of roasted-aroma compounds (Zhan et al., 2023).

In the present study, the OAV of 2-ethyl-5-methyl-pyrazine increased progressively from 1.67 ± 1.47 at RGZS to 74.92 ± 1.90 at RG1O, 216.42 ± 12.49 at RG2O, and 265.44 ± 62.88 at RG3O. As a typical Maillard-derived volatile, it may have become an increasingly important potential contributor to the nutty and roasted aroma characteristics of Rougui during the later roasting stages. The OAV of *p*-cymene, rather than *p*-menthane, increased to 167.05 ± 3.70 at RG3O, suggesting its potential contribution to citrus-like and aromatic complexity. Similarly, (+)-dihydrocarvone reached an OAV of 172.68 ± 35.48 and may have contributed to warm, herbal, and spearmint-like aroma characteristics Methyl phenylacetate increased to 47.44 ± 0.66 at RG3O, which may be associated with floral, honey-like, and sweet aroma attributes. In contrast, *p*-tolyl acetate reached an OAV of 93.34 ± 19.01 and was more closely associated with narcissus-like and phenolic nuances than with honey-like sweetness.

Overall, among the 17 core differential volatiles, 2-ethyl-5-methyl-pyrazine, (*E, E*)-2,4-heptadienal, (+)-dihydrocarvone, and *p*-cymene exhibited distinct stage-dependent OAV patterns and represented potential aroma-active compounds. Their coordinated changes were associated with the transition of Rougui aroma characteristics from a fresh, citrus–herbal, and floral–fruity profile toward a more mature roasted and nutty character. However, these compounds should be regarded as potential contributors to, rather than sole determinants of, the overall aroma transformation.

### 3.4 Characterization of non-volatile metabolites in Rougui at different roasting stages

#### 3.4.1 Composition of non-volatile metabolites and discrimination analysis

**Figure 5:**
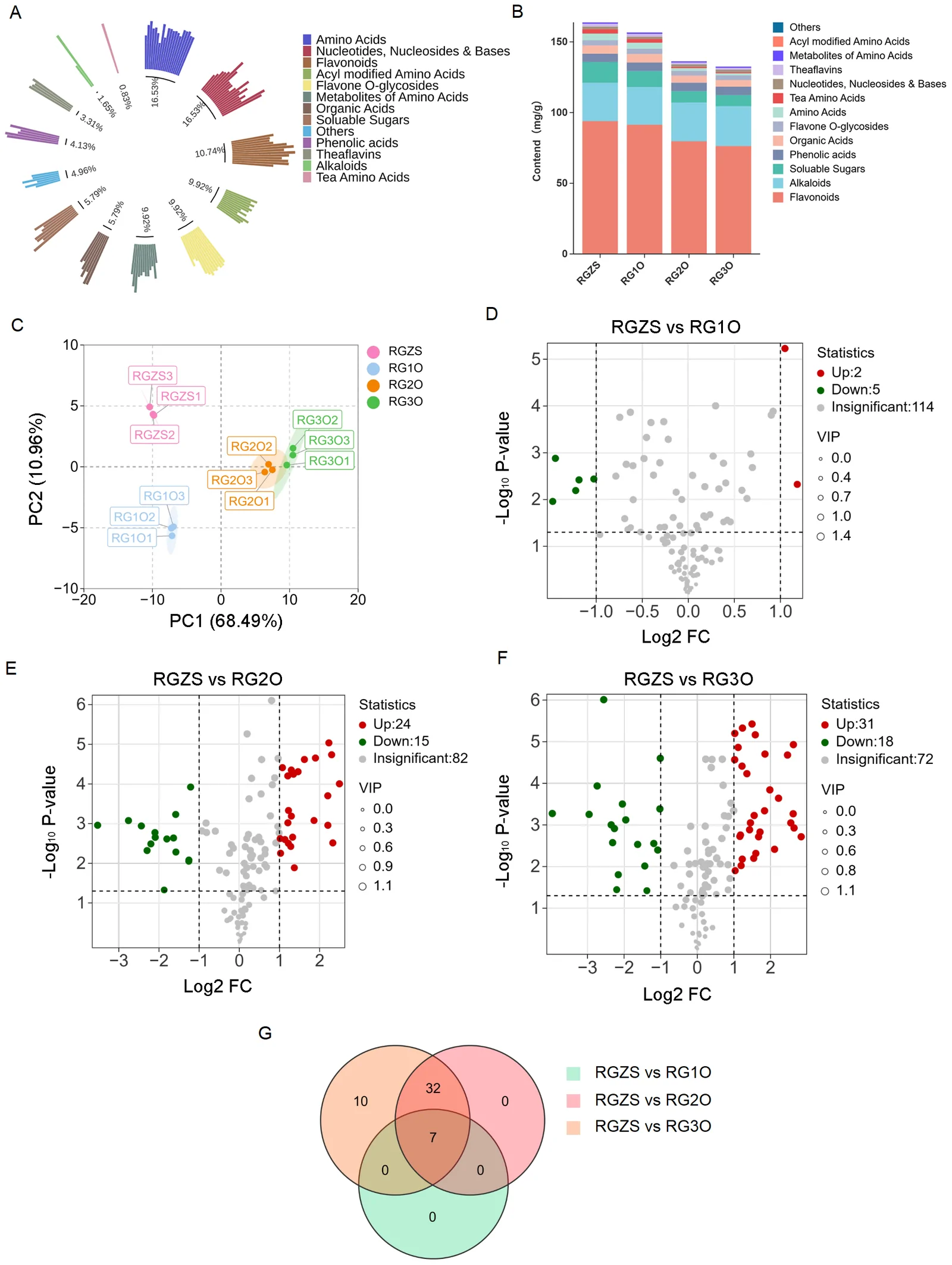
Multivariate statistical analysis of non-volatile metabolites in Wuyi Rougui. **(A)** Classification statistics of non-volatile metabolites. **(B)** Stacked bar chart showing the relative abundances of different metabolite classes. **(C)** Principal component analysis (PCA) of Rougui samples. **(D–F)** Volcano plots of RG1O, RG2O, and RG3O compared with RGZS. Differential metabolites were identified based on p< 0.05 and log_2_ fold change thresholds. **(G)** Venn diagram of RG1O, RG2O, and RG3O versus RGZS comparison groups.

Non-volatile metabolites constitute the chemical foundation of tea taste and mouthfeel (Liu et al., 2022). Wuyi Rougui is renowned for its full-bodied, mellow taste, strong sweet aftertaste, and distinctive “rock charm” character, yet the material basis underlying its thick mouthfeel remains largely unexplored (Wang et al., 2024; Ye et al., 2025). Here, a targeted metabolomic approach was adopted to systematically characterize the dynamic changes of non-volatile compounds in Rougui throughout the roasting process, with the aim of elucidating the molecular mechanisms driving taste reconstruction. A total of 121 non-volatile metabolites were detected across the four roasting stages and classified into 13 chemical categories (**Figure 5A**). Amino acids and nucleotides, nucleosides, and bases were the two most diverse categories, each comprising 20 compounds (16.53%), followed by flavonoids with 13 compounds (10.74%). Acyl-modified amino acids, flavone O-glycosides, and metabolites of amino acids each contained 12 compounds (9.92%). Organic acids and soluble sugars each comprised seven compounds (5.79%), whereas phenolic acids, theaflavins, alkaloids, and tea amino acids comprised five (4.13%), four (3.31%), two (1.65%), and one (0.83%) compounds, respectively. The remaining six compounds (4.96%) were grouped into the “Others” category. These chemically diverse metabolites collectively constitute the non-volatile chemical basis of the taste and mouthfeel characteristics of Wuyi Rougui tea. Quantitative analysis showed that flavonoids, mainly catechins, together with alkaloids consistently dominated the non-volatile metabolite profile, accounting for more than 70% of the total content. As roasting progressed, the total content of the detected non-volatile metabolites progressively decreased, indicating an overall thermal depletion pattern (**Figure 5B**). Soluble sugars decreased markedly from 14.65 mg/g in RGZS to 7.93 mg/g in RG3O. Nitrogen-containing metabolites exhibited similar decreasing trends: theanine content decreased from 3.11 mg/g to 0.67 mg/g, while the combined content of the other amino acids decreased from 4.66 mg/g to 1.49 mg/g. This depletion may be associated with their consumption through Maillard reactions and their conversion into precursors of roasted aroma compounds (Shi et al., 2023). Meanwhile, the content of flavone O-glycosides decreased moderately from 3.76 mg/g to approximately 3.31 mg/g. These changes may reflect the differential thermal stability and transformation of non-volatile metabolites under increasing roasting intensity (Peng et al., 2024). In particular, the moderate degradation of flavonoid derivatives may have contributed to reduced astringency and the gradual transition of the taste profile from raw and harsh to mellow.

Principal component analysis of quantitative metabolite data revealed distinct stage-specific clustering of samples (**Figure 5C**). The first two principal components explained 68.49% (PC1) and 10.96% (PC2) of variance, respectively, with a cumulative contribution of 79.45%. Samples were clearly separated along PC1, suggesting that roasting-associated changes contributed substantially to the differentiation of metabolite profiles among different stages. Notably, RG2O and RG3O samples clustered independently but were relatively close along PC1, suggesting high similarity in overall metabolic phenotypes. Based on *p* < 0.05 and log_2_ fold change (Log_2_FC), the 121 metabolites were further analyzed for significant alterations across comparisons. Relative to RGZS, the number of significantly up- and down-regulated compounds increased with roasting cycles (**Figures 5D–F**). RGZS vs RG3O showed the greatest number of altered compounds, including significantly upregulated metabolites such as glutamine, glutamic acid, and *D*-fructose, and significantly downregulated compounds such as cytosine, *N*-acetylalanine, and myricetin. These results suggest that prolonged roasting was associated with extensive remodeling of the non-volatile metabolite profile, involving changes in nitrogen-containing metabolites, sugars, and flavonoid-related compounds. Among comparisons, RGZS vs RG1O exhibited the smallest changes (**Figure 5D**), with only rhamnose and gallocatechin significantly upregulated, while *N*-acetylalanine, cytosine, *N*6-methyladenosine, 4-pyridoxic acid, and pyruvic acid were significantly downregulated, suggesting that the overall alteration of non-volatile metabolite composition was relatively limited during the initial roasting stage. Venn diagram analysis of the comparison groups revealed that RGZS vs RG3O contained the largest number of unique compounds, indicating greater differences in metabolite composition between the initial and final roasting stages (**Figure 5G**). Seven differential compounds were common across all three comparisons, including *N*-acetylalanine, cytosine, *N*6-methyladenosine, pyridoxic acid, pyruvic acid, rhamnose, and gallocatechin, representing metabolites that showed consistent responses to thermal processing and may serve as potential indicators of roasting-associated metabolic changes in Rougui.

#### 3.4.2 Characteristics of non-volatile compounds and DOT analysis

**Figure 6:**
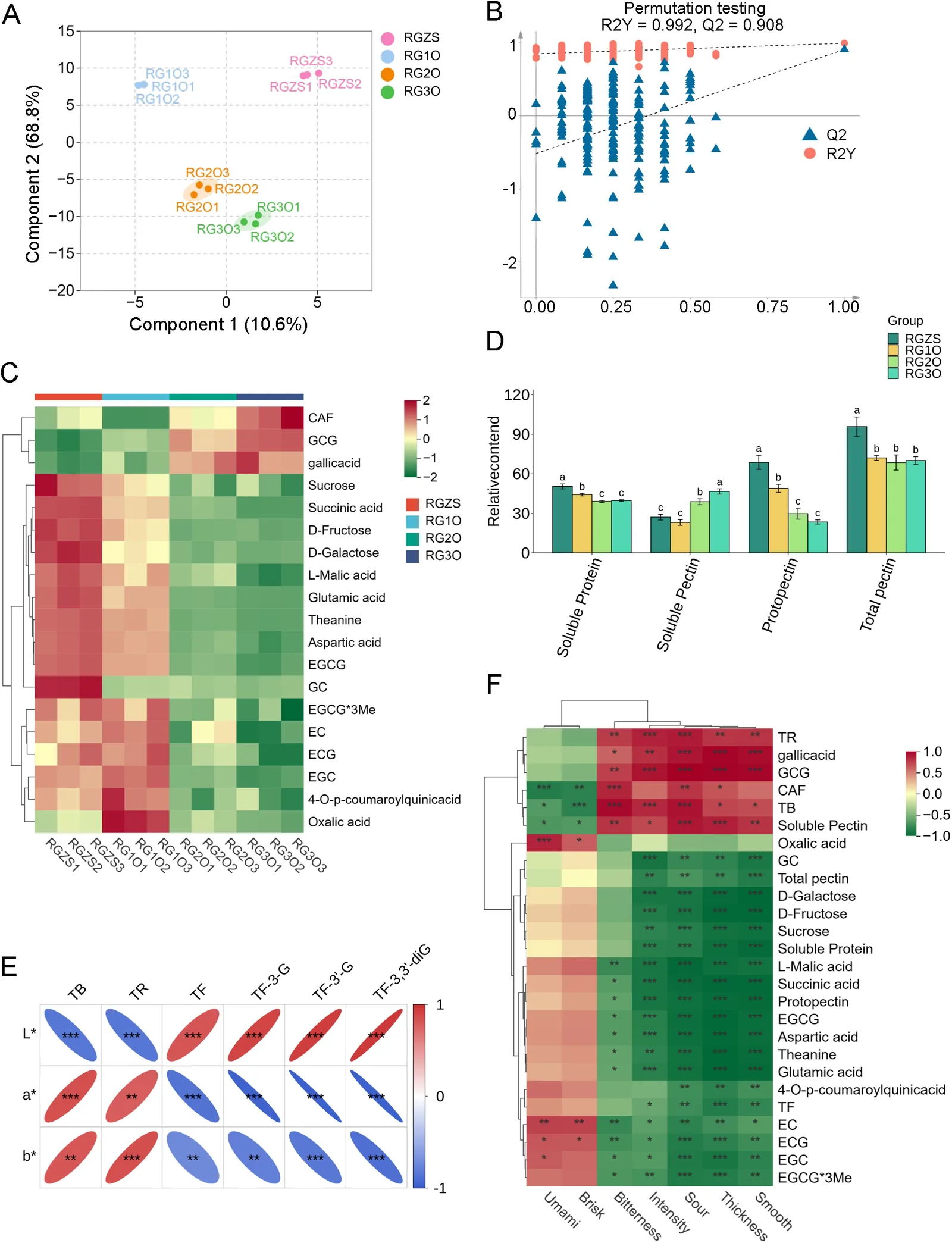
Differential analysis of non-volatile metabolites and physicochemical properties in Wuyi Rougui. **(A)** OPLS-DA analysis of Rougui samples from different roasting stages. **(B)** Permutation test results. **(C)** Clustered heat map of differential metabolites across groups. **(D)** Quantification of proteins and pectin components among groups. **(E)** Correlation heat map between tea pigment components and color parameters. **(F)** Correlation analysis between differential metabolites and sensory attributes. Data are presented as mean ± SD (n = 3). Statistical significance was determined using one-way ANOVA followed by Tukey’s HSD multiple comparison test. * indicates p < 0.05, ** indicates p < 0.01, and *** indicates p < 0.001. Different lowercase letters indicate significant differences at p < 0.05.

OPLS-DA analysis of the 121 non-volatile metabolites effectively distinguished samples from different roasting stages (**Figure 6A**). Permutation tests indicated R²Y = 0.992, Q² = 0.908, with a Q² intercept of approximately −0.5, suggesting good model fitting and predictive performance for distinguishing metabolite profiles among different roasting stages. (**Figure 6B**). Using VIP > 1.0 and *p* < 0.05 as thresholds, 19 core differential metabolites were identified. These included key taste-related compounds such as catechin monomers and their isomers, alkaloids, phenolic acids, and amino acids. The VIP values of gallocatechin (GC), epigallocatechin (EGC), and epigallocatechin gallate (EGCG) were 5.14, 4.79, and 3.17, respectively, reflecting major chemical differences before and after roasting. GA showed a VIP of 1.11 and was identified as one of the differential metabolites associated with roasting-related quality changes. Hierarchical clustering visualized in a heatmap revealed the trends of these 19 key differential metabolites along the roasting gradient (**Figure 6C**). Two evolution patterns were observed. The first pattern exhibited significant thermal depletion, encompassing most catechin monomers, amino acids, and soluble sugars. For instance, EGC decreased from 28.43 mg/g at RGZS to 21.53 mg/g at RG3O, GC decreased from 6.49 mg/g to 2.76 mg/g, and EGCG decreased from 38.31 mg/g to 32.68 mg/g. Theanine exhibited pronounced thermal degradation, with its content decreasing from 3.11 mg/g to 0.67 mg/g. At the same time, sugars such as sucrose, *D*-galactose, and *D*-fructose also showed marked decreases. The reduction of these compounds may contribute to the attenuation of astringency and the gradual transition of tea taste toward a smoother sensory profile. However, the contribution of individual metabolites to taste perception likely depends on their concentrations, interactions, and sensory thresholds within the tea matrix (Lu et al., 2024; Su et al., 2024). The second pattern exhibited thermal-induced accumulation, represented by GA and GCG. GA content increased steadily with roasting, from 1.24 mg/g at RGZS to 1.93 mg/g at RG3O, while GCG increased from 0.56 mg/g to 1.13 mg/g. These dynamic changes suggest possible transformation of catechin-related compounds during roasting.

To evaluate the sensory contribution of these chemical changes, the DOT approach was applied using a 1:22 brewing ratio (**Supplementary Table 5**). Results showed that although the content of esterified catechins decreased with roasting, their DOT values remained high. As representative compounds of raw astringency and initial freshness, the DOT values of GC and glutamic acid fell below the threshold by the RG1O stage. In contrast, the DOT value of GA increased from 1.65 to 2.57. Notably, although the DOT value of GCG remained below 1.0, it exhibited a continuous accumulation trend with increasing roasting intensity. Although GCG showed a DOT value below 1.0, its continuous accumulation during roasting suggests that it may participate in the overall taste profile through potential interactions with other taste-active compounds. Therefore, GCG, together with GA and other catechin derivatives, may contribute to the enhanced fullness and persistence of tea taste. Recent studies based on deletion and reconstitution experiments have suggested that esterified catechins may play important roles in shaping the taste characteristics of Rougui and other oolong teas (Yu et al., 2025). To investigate the material basis of liquor thickness, protein and pectin dynamics during roasting were analyzed (**Figure 6D**). Insoluble protopectin decreased significantly with roasting, by 65.8%, whereas water-soluble pectin increased by 72.0%. Soluble protein content declined from 50.45 mg/g to 39.77 mg/g, a decrease of approximately 21.2%. These results indicate that the thermal environment induced protein denaturation and physicochemical transformations of pectin, which may alter the molecular characteristics of pectin and contribute to changes in viscosity and mouthfeel properties of the tea infusion. Similar thermal-induced *β*-elimination degradation of cell wall polysaccharides, which may contribute to increased water-soluble pectin levels, has been reported in green tea and plant-based foods such as hawthorn (Hirono and Mizukami, 2018; Zhou et al., 2021).

Furthermore, to elucidate the co-regulation of liquor color and taste evolution, the correlation between tea pigments and color parameters was analyzed (**Figure 6E**). Correlation matrices showed that theabrownins and thearubigins were significantly negatively correlated with L^*^ and positively correlated with a^*^ and b^*^, indicating that TBs accumulation was closely associated with changes in the liquor color from bright to deep red. The 42.0% loss of thearubigins during roasting accounts for the fading of yellow-green tones. The thickening effect of high-molecular-weight TBs, combined with color changes, may contribute to the sensory quality transformation of Rougui during roasting. Correlation heatmap analysis further revealed intrinsic links between metabolite changes and sensory attributes (**Figure 6F**). The contents of water-soluble pectin, GA, GCG, and TBs were positively correlated with Thickness and Smoothness, and negatively correlated with Bitterness and Astringency. These dynamic changes were associated with the transition of Rougui taste from raw astringency to sweetness and fullness, with thermal conversion potentially contributing to the modification of taste characteristics.

### 3.5 Molecular docking analysis of key aroma and taste compounds

Molecular docking offers computational predictions of potential ligand–receptor interactions through molecular simulation approaches (Shen et al., 2025). To further explore the potential molecular interactions underlying aroma and taste perception in roasted Rougui, potential aroma and taste compounds were docked with the olfactory receptor OR1A1 and the calcium-sensing receptor CaSR, respectively. The selection of specific volatile ligands was primarily based on their odor activity values (OAVs) and dynamic variation patterns during the roasting stages (**Supplementary Table 4**). Based on their OAVs and dynamic changes during roasting, six volatile compounds were selected to represent different sensory attributes: 1-nonen-3-one and indole, which consistently exhibited high OAVs; 2-methylpropyl pyrazine and 5-ethyl-2-methyl-pyridine, whose OAVs steadily increased during roasting and were considered potential aroma-active compounds associated with roasted characteristicsa; and hotrienol and (*E*, *E*)-2,4-heptadienal, which were associated with floral and green aroma characteristics.

**Figure 7:**
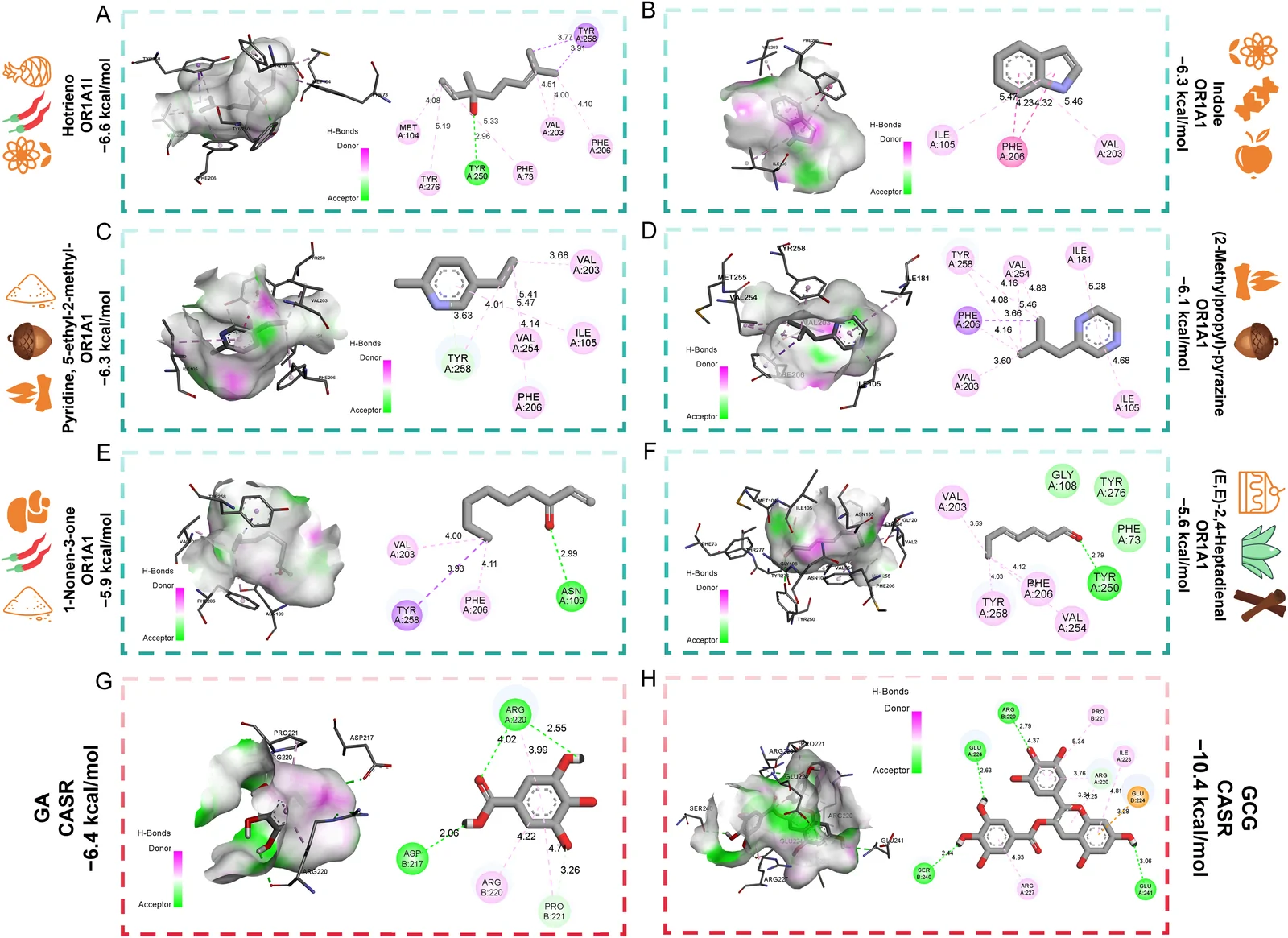
Molecular docking simulation of selected flavor compounds. **(A–F)** Predicted binding modes of core volatile aroma compounds with the olfactory receptor OR1A1: dehydro-linalool, indole, 5-ethyl-2-methylpyridine, 2-methylpropyl pyrazine, 1-nonen-3-one, and (*E*, *E*)-2,4-heptadienal, respectively. **(G, H)** Three-dimensional docking and amino acid interactions of non-volatile metabolites with the calcium-sensing receptor (CaSR): gallic acid (GA) and gallocatechin gallate (GCG), respectively.

Their interactions with the broadly tuned human olfactory receptor OR1A1 were simulated. As shown in **Figure 7**, all ligands exhibited negative binding energies ranging from -5.6 to -6.6 kcal/mol, indicating predicted favorable interaction tendencies between ligands and OR1A1. Among them, hotrienol (dehydro-linalool) showed the highest predicted affinity for OR1A1, with a binding energy of -6.6 kcal/mol (**Figure 7A**). This molecule formed a hydrogen bond with Tyr250 and was further stabilized within the binding pocket through hydrophobic interactions with Met104, Tyr276, and Phe73, which suggests a possible interaction mode between hotrienol and OR1A1 that may be related to its potential aroma activity. In comparison, 2-methylpropyl pyrazine, a roasted-aroma-associated compound, exhibited a favorable binding energy of -6.1 kcal/mol (**Figure 7D**). Given its high OAV, 1-nonen-3-one may represent a potential aroma-active compound, and molecular docking showed that it interacted with Asn109 through hydrogen bonding (**Figure 7E**). 5-Ethyl-2-methylpyridine was further stabilized by a carbon–hydrogen bond with Tyr258 (**Figure 7C**). Indole (**Figure 7B**) and (*E*, *E*)-2,4-heptadienal (**Figure 7F**) primarily relied on hydrophobic interactions with Ile105, Val203, and Phe206 to fit within the receptor-binding pocket. These findings are consistent with the GC-MS and QDA sensory results, suggesting that roasting-induced remodeling of aroma compounds may be associated with changes in aroma perception through potential ligand–receptor interactions.

Regarding taste perception, the interactions between selected taste-related compounds in Rougui and the calcium-sensing receptor were further simulated. GCG exhibited the highest predicted binding affinity, with a Vina score of -10.4 kcal/mol, and established an extensive hydrogen-bonding network within the binding pocket (**Figure 7H**), suggesting a a potentially stable interaction with CaSR in the docking model. This finding is consistent with the positive correlation between GCG abundance and sensory thickness scores observed in the correlation analysis (r = 0.962). Meanwhile, GA, a major roasting-associated derivative of catechin-related compounds, also displayed favorable binding with CaSR, with a binding energy of -6.4 kcal/mol (**Figure 7G**). GA formed multiple hydrogen bonds with Arg A:220, Asp B:217, and Pro B:221 and was further stabilized in the binding site by π-alkyl interactions with Arg B:220. Despite its relatively small molecular size, the increase in the DOT value of GA from 1.66 to 2.58 during roasting suggests an increased potential contribution to taste perception during roasting. The combined effects of GCG and GA may be associated with the overall modification of taste characteristics during roasting. Docking analyses provide additional predictive evidence for the potential interaction between these compounds and CaSR, although their actual contribution to kokumi-related perception requires further sensory and receptor-based validation. Together with the physical thickening effects of macromolecules such as pectin, this integrated evidence suggests that both macromolecular changes and small-molecule interactions may contribute to the transformation of Rougui tea taste toward a smoother and fuller-bodied sensory profile with a sweet aftertaste.

## 4 Conclusion

This study provides an integrated characterization of flavor reconstruction in Wuyi Rougui tea during roasting. The RG2O stage showed favorable sensory characteristics with enhanced fruity-floral and cinnamon-like aroma attributes. Flavor evolution was associated with the coupled transformation of volatile and non-volatile metabolites, while liquor thickness may involve the combined effects of the remodeling of key taste-active metabolites and the structural conversion of pectin. These findings highlight the integrated roles of chemical transformation and physical modification in shaping tea quality and provide insights into the potential basis for optimizing roasting processes in oolong tea.

## Data Availability

The original contributions presented in the study are included in the article/**Supplementary Material**. Further inquiries can be directed to the corresponding authors.
