# Supplementary material for: Roasting-driven flavor reconstruction in Wuyi Rougui tea: a dual mechanism of pectin-mediated thickening and metabolite remodeling
Source: Front Plant Sci. 2026 Aug 28;17:1938406. doi: 10.3389/fpls.2026.1938406 (PMC13561936; doi:10.3389/fpls.2026.1938406)
Supplement: Supplementary file 1 [file DataSheet1.docx]

**Supplementary Material**

**Title:** Roasting-driven flavor reconstruction in Wuyi Rougui tea: a dual mechanism of pectin-mediated thickening and metabolite remodeling

**1. Reagents and chemicals**

**(1) Determination of catechins, caffeine, theobromine, amino acids, nucleotides, choline, betaine, organic acids and soluble sugars, flavonols, glycosides, phenolic acids, and theaflavin components**

Mass spectrometry-grade methanol, acetonitrile, formic acid, ammonium formate, ammonium acetate, and isopropanol were purchased from Fisher Scientific (Fair Lawn, USA). Ultrapure water (18.2 MΩ·cm) was prepared using a Milli-Q water purification system (Merck Millipore, USA). HWS-28 electric thermostatic water bath (Haiyiheng Scientific Instrument Co., LTD.); 3K15 SIGMA Medical centrifuge (Xima Centrifuge (Yangzhou) Co., LTD.); ME204E Electronic Balance (METTler). C18 reversed phase column (4.6*250 mm, 5 μm) (Thermo Fisher Technology (China) Co., LTD.); Nylon 6 needle filter (0.45 μm, Guangzhou Green Baicao Scientific Instrument Co., LTD.) EDTA-Na2, ascorbic acid and chemical derivatization reagents, including 3-nitrophenylhydrazine hydrochloride, 1-ethyl-(3-dimethylaminopropyl) carbodiimide hydrochloride, and pyridine, were obtained from Sigma‒Aldrich (Merck KGaA, Darmstadt, Germany).

The standard substance information is integrated by manufacturer as follows: Shanghai Yuanye Biotechnology Co., Ltd. provides the following standards: Gallocatechin (GC, CAS: 3371-27-5, specification: 20mg, HPLC≥98%), Epigallocatechin (EGC, CAS: 970-74-1, specification: 20mg, HPLC≥98%), Catechin (C, CAS: 154-23-4, specification: 100mg, HPLC≥98%), Theophylline (TP, CAS: 58-55-9, specification: 50mg, HPLC≥98%), Epigallocatechin Gallate (EGCG, CAS: 989-51-5, specification: 20mg, HPLC≥98%), Epicatechin (EC, CAS: 490-46-0, specification: 20mg, HPLC≥98%), Gallocatechin Gallate (GCG, CAS: 4233-96-9, specification: 20mg, HPLC≥98%), Epigallocatechin-3-O-(3-O-methyl)gallate (3-O-Methyl-EGCG, CAS: 83104-87-4, specification: 5mg, HPLC≥98%), Epicatechin Gallate (ECG, CAS: 1257-08-5, specification: 20mg, HPLC≥98%), and Catechin Gallate (CG, CAS: 130405-40-2, specification: 20mg, HPLC≥98%). Beijing Tanmo Quality Inspection Technology Co., Ltd. supplies Theobromine (TB, CAS: 83-67-0, specification: 100mg, purity 99.3%). Beijing Manhage Biotechnology Co., Ltd. provides Caffeine (CAF, CAS: 58-08-2, specification: 999.7 μg/mL).

Nonisotopically labelled external standards were purchased from Sigma‒Aldrich (Merck KGaA, Darmstadt, Germany), Macklin Inc. (Shanghai, China), Toronto Research Chemicals (Toronto, Canada), Aladdin Biochemical Technology Co., Ltd. (Shanghai, China), MedChemExpress LLC (Shanghai, China), Tokyo Chemical Industry Co., Ltd. (Tokyo, Japan), J&K Scientific (Beijing, China), CAYMAN Chemical (Michigan, USA), and other companies. Stable isotopically labelled internal standards were obtained from Toronto Research Chemicals (Toronto, Canada), Cambridge Isotope Laboratories (Andover, USA), Sigma‒Aldrich (Merck KGaA, Darmstadt, Germany), Macklin Inc. (Shanghai, China), and other companies.

**(2) Detection of Thearubigins and Theabrownins**

Oxalic acid (CP, 500 g, Tianjin Guangfu Technology Development Co., Ltd.); ethanol (AR, 500 mL, Xilong Scientific Co., Ltd.); n-butanol (AR, 500 mL, Tianjin Yongda Chemical Reagent Co., Ltd.); ethyl acetate (HPLC, 4 L, OCEANPAK); and sodium bicarbonate (CP, 500 g, Tianjin Guangfu Technology Development Co., Ltd.) were used.

**2. Supporting Information Tables**

(Note: Please refer to the separate Excel files for full datasets)

Table S1. Names and performance of sensors in the electronic nose.

Table S2. Odor activity values (OAVs) and odor descriptions of 76 differential volatile compounds (VIP > 1) in Wuyi Rougui tea during different roasting stages.

Table S3. Odor activity values (OAVs) of 17 core differential volatile compounds consistently altered across all roasting stages in Wuyi Rougui tea.

Table S4. Taste thresholds and dose-over-threshold (DOT) values of core non-volatile compounds in Wuyi Rougui tea during different roasting stages.
